# Sustainability and cost comparison of 33 kV overhead transmission lines and underground cables: a life cycle perspective

**DOI:** 10.1038/s41598-026-48702-0

**Published:** 2026-04-21

**Authors:** MD. Faiyaz Ahmed, Md Atiqur Rahman, Prabhu Paramasivam, Mohamed Yusuf, S. M. Mozammil Hasnain

**Affiliations:** 1 Department of Mechanical Engineering, Vignan’s Foundation for Science Technology and Research, Guntur, India; 2 Department of Research and Innovation, Saveetha School of Engineering, SIMATS, Chennai, Tamil Nadu 602105 India; 3 Department of Peace and Development Studies, Njala University, Bo Campus − 18, Bo, Sierra Leone; 4 Department of Mechanical Engineering, Faculty of Engineering & Technology, Marwadi University Research Center, Marwadi University, Rajkot, Gujarat 360003 India

**Keywords:** Life cycle assessment, Environmental impact, Electric cable, Underground cables, Cost analysis, Climate sciences, Energy and society, Energy science and technology, Engineering, Environmental sciences

## Abstract

This Manuscript presents an integrated Life Cycle Assessment (LCA) and Life Cycle Costing (LCC) evaluation of five 33 kV distribution designs, including three overhead (OH) lines and two underground (UG) cable systems, under typical Indian operating circumstances. All environmental and economic impacts are constantly normalized per supplied kWh during a 40-year service life, allowing for clear techno-environmental comparisons. The results show that global warming potential (GWP), fossil resource depletion and particulate matter generation are the most significant environmental effect categories. Operational I²R conductor losses are the largest contributor to lifespan impacts when analyzed per delivered electricity. This highlights the relevance of conductor resistance and loading circumstances in infrastructure planning. The comprehensive study demonstrates a clear cost-environment trade-off: underground systems have larger embodied material impacts and capital expenditure, whereas overhead systems have lower initial impacts but are still susceptible to operational loss-related consequences over time. The prevalence of operational losses is exacerbated by Indian grid emission factors and loading practices, emphasizing the significance of region-specific lifecycle normalization. The study establishes a decision-relevant paradigm for assessing 33 kV distribution infrastructure by explicitly connecting conductor design, lifespan environmental performance, and long-term cost efficiency. These insights help utilities and governments optimize medium-voltage infrastructure for ecological and economically effective power delivery.

## Introduction

In India, 33-kV electric power distribution networks serve as a vital interface between transmission systems and end-use users. As urbanization, industrial expansion and infrastructure development continue, the choice of suitable distribution infrastructure has a considerable impact on long-term energy efficiency, dependability, lifetime cost and environmental performance. Decisions taken at this voltage level are especially important since distribution networks function continuously for multiple decades and account for significant material consumption and technical losses^1^. 33 kV distribution systems use two main configurations: overhead (OH) lines and underground (UG) cable systems. Overhead systems have reduced installation costs, easier maintenance, and simplified heat dissipation, but they are more vulnerable to environmental disturbances and faults. Underground systems have benefits such as increased reliability in dense metropolitan areas, decreased visual effect and protection against weather-related damage, but they necessitate more material, insulation systems, trenching operations, and difficult installation methods. These structural and operational variances have a direct impact on capital investment, operating losses, embodied emissions, and long-term maintenance costs^2^. As a result, robust technoeconomic-environmental comparisons are required for informed infrastructure development. Previous research has examined particular facets of this comparison. Subterranean systems demand a substantially larger initial capital expenditure than overhead lines, according to cost-focused research. Reduced outage frequency in subterranean networks is emphasized by reliability-oriented evaluations, especially in metropolitan environments. Power transmission infrastructure lifecycle assessment (LCA) studies show that conductor materials like copper and aluminum account for the majority of embodied emissions. Nevertheless, the majority of earlier studies normalize environmental effects per kilometer of line instead of per delivered electricity, which makes it difficult to compare configurations with various loads and loss characteristics. Moreover, integrated lifetime evaluation is hindered by the frequent separation of operational I²R transmission losses from embodied consequences^3^. There are few comprehensive studies that include lifespan environmental consequences, lifecycle cost assessment (LCC), and operational loss modeling for medium-voltage (33 kV) systems, particularly under Indian conditions.

According to this review, three important deficiencies can be found. First, there is a lack of a uniform functional unit based on supplied electricity, which is required for consistent comparisons of different distribution technologies. Second, previous research has not fully addressed the incorporation of operational transmission losses into lifecycle environmental normalization. Third, comparable assessments rarely include regional-specific data on Indian conductor diameters, loading practices, installation procedures, and service lifetimes. These constraints impede effective infrastructure decision-making for developing and quickly expanding distribution networks.

### Goal and scope

This study addresses these gaps by presenting a comprehensive India-specific comparison of 33 kV overhead transmission lines and underground cable systems using an integrated Life Cycle Assessment (LCA) and Life Cycle Costing (LCC) framework. The primary functional unit is defined as the delivery of 1 kWh of electricity over a 40-year service life, enabling consistent normalization of both environmental impacts and economic costs. The analysis explicitly incorporates operational I²R losses into the lifecycle model, thereby linking conductor design, system configuration and energy performance to environmental outcomes. Environmental indicators including global warming potential, particulate matter formation and fossil resource depletion are evaluated alongside capital, operation, maintenance and end-of-life costs. Sensitivity analyses reflecting Indian operating conditions such as load variation and installation context are further included to enhance practical relevance as shown in Table 1. The resulting framework provides a transparent and methodologically consistent basis for sustainable 33 kV distribution infrastructure planning in India.

**Table 1:** Conductor size and maximum allowable loading.

Size of conductor	Absolute max loading (A)	Max loading normal (A)
25 mm^2^ overhead lines (copper)	207	105.7
38 mm^2^ overhead lines (copper)	264	132.3
100 mm^2^ overhead lines (copper)	485	245
95 mm^2^ underground (aluminium)	285	143.6
185 mm^2^ underground (aluminium)	411	205.6

## Assumptions and methodology

### Life cycle inventory (LCI) and life cycle impact assessment (LCA) approach in Indian context

The Life Cycle Inventory (LCI) and Life Cycle Impact Assessment (LCA) methodologies adopted in this study are tailored to the Indian context to ensure relevance and accuracy. Authors assume uniform geographic and climatic conditions typical of India, considering standard practices and regulations in the Indian power sector. Both 33 kV overhead transmission lines and underground cables are evaluated over an operational lifespan of 40 years. The LCI phase involves collecting data on raw material extraction, manufacturing, transportation, installation, operation, maintenance and end-of-life disposal. This data is specific to Indian suppliers and construction practices, including the types of materials commonly used and the energy mix for manufacturing and operational processes.

### Methodology

The LCA methodology follows the ISO 14,040 and ISO 14,044 standards, incorporating environmental impact categories such as global warming potential, acidification potential and resource depletion. The analysis compares the two systems by quantifying their environmental impacts from cradle to grave ^4,5^,. For the cost analysis, authors consider initial capital expenditure, operational and maintenance costs and decommissioning costs, adjusted for local economic conditions, inflation rates and government subsidies or incentives specific to India. Sensitivity analyses are conducted to account for regional variations in material availability, labor costs and energy prices. This comprehensive approach ensures that the assessment reflects the unique challenges and opportunities of deploying 33 kV transmission lines and underground cables in India.

### Maximum normal cable loading

The maximum normal cable loading for 33 kV electrical transmission lines and underground cables is critical for ensuring efficient and reliable power delivery. For 33 kV overhead transmission lines, designed to handle high ambient temperatures typical of India, current loads generally range from 500 to 800 Amperes under normal conditions, considering factors like conductor material (aluminum or aluminum alloy), ambient temperature, wind speed and solar radiation. For 33 kV underground cables, insulated with cross-linked polyethylene (XLPE) and installed in conditions where high soil temperatures are common, the loading capacity is typically between 600 and 900 Amperes. Effective heat dissipation through proper cable trench design, including adequate spacing and thermal backfill materials, is essential to maintain performance. Both types of installations must consider the highest expected ambient and soil temperatures, utilize appropriate cooling mechanisms and follow proper installation practices to avoid overheating and ensure reliable operation.

### Conductors with constant temperature in the Indian environment

In India’s diverse and challenging climate, constant temperature conductors (HTLS conductors) offer a significant advantage for the transmission of electricity over traditional conductors. These conductors can operate at temperatures up to 210 °C or higher, compared to the 75 °C to 90 °C limits of conventional conductors. This increased temperature tolerance allows for higher current carrying capacities, making them suitable for upgrading existing transmission lines without the need for major infrastructure changes. In India, where the demand for electricity is continuously rising, HTLS conductors can help manage the increasing load by allowing more power to be transmitted through the same physical infrastructure. The unique design of HTLS conductors, which includes advanced core materials such as composite cores or aluminum composite materials, minimizes sag at elevated temperatures, ensuring consistent performance and reliability. This is particularly beneficial in areas with high ambient temperatures and difficult terrain, where maintaining conductor clearance is crucial. By incorporating HTLS technology, India can enhance the efficiency and reliability of its electrical grid, support the integration of renewable energy sources and reduce the environmental impact of transmission lines.

### Demand of load

In India, load demand is a dynamic variable that varies daily, seasonally, and 365 days a year. It also changes hourly. The “load factor” is defined as the ratio of the actual kilowatt-hours transferred over the course of the year to the kilowatts at peak demand over 8760 h (one year). The power distribution firm of Andhra Pradesh, India, performed a thorough study of load demands every half-hour during the 2008/9 period of operation, and the results showed a load factor of 0.679. As a result, a “loss load factor” is used to indicate losses, which are determined by the square of the load current. This is the load factor squared, or 0.679², which comes to 0.461. A steady and dependable power supply, a reduction in transmission losses, and the advancement of India’s goals for energy security and economic growth all depend on the effective management of these load demands.

### Nation-specific adaptations

Materials for 33 kV electrical transmission lines and subterranean cables in India are acquired from several nations, guaranteeing a reliable and superior infrastructure. Indian and Chinese steel, Canadian lumber, Australian aluminium, and Indian copper were utilized, illustrating the commodities’ varied supply chain. The underlying data for these materials was modified to take into account the electrical combinations unique to each nation that are pertinent to the Indian setting. Australia, Canada and China’s electrical markets were covered, which provided reliable Life Cycle Inventory (LCI) data. Using data from the International Energy Agency (IEA), the electrical generation mix for India was explicitly estimated and then updated to account for regional differences and the unique features of the Indian infrastructure. This method guarantees that, in the context of India, the environmental impact assessment appropriately captures the emissions and power usage related to material production and use^6–8^. Equation 1 may be used to determine the energetic quality of heat.

1 $$\:\theta\:=1-\frac{{T}_{o}}{{T}_{p}}$$

where $\:\theta\:=$ Exergetic quality; $\:{T}_{o}$ = Ambient temperature, K, $\:{T}_{p}\:$ = Process temperature, K.

For the heat produced by district Combined Heat and Power (CHP) systems, an exergy quality factor of 0.2 was determined using Eq. 1. This estimate was produced using operational parameters unique to the Indian context: a 70 °C delivery water temperature and an average ambient temperature of 0 °C. An accurate evaluation of the heat produced energy efficiency is made possible by these characteristics, which represent typical operating circumstances for district CHP systems. Taking into account the entire production less internal energy sector consumption and distribution losses, the exergy quality parameters were applied to the total energy generated and distributed to consumers in India. It was calculated that 25.5% of all fuel inputs used in the production of electricity in India went toward heat, even in the case of hydroelectric power, which requires no thermal input. In particular, the allocation factors for the primary technologies were as follows: 53% for oil, 34% for gas-generated electricity, and 30% for heat for coal (meaning that 70% of the fuel is given to electricity). These variables, which are specific to the Indian energy production setting, consider the heat-to-power ratios of the corresponding technologies as well as the quality of the energy.

### Installation

The installation of 33 kV electrical transmission lines and underground cables in India involves meticulous planning and execution tailored to local conditions and regulatory requirements. For overhead transmission lines, the process begins with route surveys to identify the most efficient path, followed by obtaining necessary land acquisition and environmental clearances. Foundations are then constructed for transmission towers, which are erected with precision to ensure stability. Conductors are strung between towers with careful tensioning to prevent sagging, and the system is rigorously tested for electrical continuity and safety. For underground cables, a detailed route survey is also essential, along with trench excavation to the required depth and width. Cables are laid in protective ducts, jointed, and terminated with meticulous sealing and insulation. The trenches are backfilled and compacted, and comprehensive tests are conducted to ensure the integrity and safety of the system^9^. Both methods prioritize safety, environmental impact minimization and compliance with local regulations, with overhead lines offering quicker and less expensive installation but greater susceptibility to environmental damage and underground cables providing greater protection and longevity despite higher costs and longer installation times. This holistic approach ensures efficient, safe and environmentally sustainable installation of electrical transmission infrastructure in India.

### Maintenance

Maintenance of 33 kV electrical transmission lines and underground cables in India is crucial to ensure reliable and efficient power delivery. For overhead transmission lines, regular inspections are conducted to check for physical damage, corrosion, and wear on conductors and towers. Vegetation management is essential to prevent tree branches from interfering with the lines. Thermal imaging and other advanced diagnostic tools are used to detect hotspots and potential points of failure. Repairs and replacements are carried out promptly to maintain system integrity, often requiring coordination with local authorities to minimize disruptions. For underground cables, maintenance involves periodic testing and monitoring of the cable insulation and joint integrity. Advanced techniques such as partial discharge testing, time-domain reflectometry and cable sheath testing are employed to identify potential faults. Since underground cables are less accessible, maintenance planning includes detailed records of cable routes and access points. Preventive maintenance also involves ensuring the integrity of cable ducts and manholes, managing water ingress, and maintaining cooling systems where applicable.

Maintenance of overhead 33 kV electrical transmission lines in India includes multiple essential activities to ensure their continued performance and safety.

- Helicopter flyover inspections are conducted every two years, with an estimated 4 min of flying time per kilometer of cable.
- Tree trimming is scheduled every five years, requiring travel up to 20 km from the base.
- Every ten years, inspections and non-invasive pole testing are carried out, involving 20 km of road travel from the base.
- Fault repairs are managed at a rate of 0.04 faults per year, with necessary travel distances up to 20 km from the base to the fault location. These maintenance procedures are critical for sustaining the operational reliability and safety of overhead transmission lines.
- Maintenance of 33 kV underground cables in India involves a more streamlined process compared to overhead lines. There are no routine inspections required for underground cables. However, fault repairs are addressed as they occur, with an estimated fault rate of 0.04 faults per year.
- Each repair may require travel up to 30 km from the base to the fault location. This maintenance approach highlights the reliability and lower maintenance needs of underground cable systems compared to overhead lines.

### Composition of conductors

The composition of 33 kV underground cables in India is meticulously designed to ensure durability, efficiency and safety. The core conductor, typically made of high-conductivity copper or aluminum, is surrounded by a semi-conductive conductor screen to distribute the electric field evenly. Primary insulation, often made of cross-linked polyethylene (XLPE) or ethylene propylene rubber (EPR), provides excellent electrical insulation and thermal resistance. This insulation is further protected by an insulation screen to manage electric fields and prevent discharge. A metallic screen, usually copper tape or wire, offers a path for fault currents and grounding. The material quantities presented in Table 2 for Systems 1–5 were derived from a combination of primary Indian utility design data (APTRANSCO, POWERGRID, UPPTCL), secondary technical sources (CIGRÉ TB 710, Western Power Distribution UK design manuals, XLPE cable datasheets), and engineering assumptions where direct values were unavailable. Each entry in Table 2 is now tagged as Primary (P), Secondary (S), or Assumed (A) to improve transparency. All materials were mapped to corresponding Ecoinvent 3.9 unit processes for LCIA modeling under the ReCiPe 2016 framework.

**Table 2:** Material Breakdown per Kilometer of Installation.

Material	System 1	System 2	System 3	System 4	System 5	Source*
Copper	800^p^	750^p^	700^s^	600^s^	580^s^	P/S
Aluminium	1200^p^	1100^p^	1000^p^	900^s^	880^s^	P/S
Steel	1400^p^	1300^p^	1250^p^	1150^s^	1130^s^	P/S
XLPE	500^s^	480^s^	450^s^	700^p^	680^p^	P/S
PVC	200^s^	190^s^	180^s^	300^p^	290^p^	P/S
HDPE	150^a^	140^a^	130^a^	250^a^	240^a^	A
Insulation screen	100^s^	95^s^	90^s^	150^p^	145^p^	P/S
Armoring	300^s^	280^s^	270^s^	450^p^	430^p^	P/S

*P, primary (India utility design data); S, secondary (CIGRÉ, WPD, literature); A, assumed (based on standard installation practices).

## Methodology for life-cycle assessments: midpoints

There are many different types of LCIA techniques, but they may be broadly divided into two groups: those that provide “endpoint” indicators and those that produce “midpoint” indicators. Without taking into account the actual or ultimate impacts they could produce; midpoint indicators evaluate the prospective benefits or consequences on the environment. For example, a midpoint indicator might provide a moderately uncertain scientifically rigorous methodology by quantifying the impacts on climate change in terms of CO_2_ equivalents (CO_2_e)^10^. Less is known, though, about the specific effects of CO_2_ emissions on the ecosystem. Conversely, endpoint indicators seek to shed light on how environmental changes really impact human health, ecosystem and resources, as a result of climate change. Endpoint indicators can add subjectivity and ambiguity to the assessment process, even while they provide a more thorough knowledge of environmental consequences. The goal of using endpoint indications is to make the understanding of complicated environmental data easier, as it might be difficult to read a large number of midpoint indicators. The selected LCIA framework for this study is the ReCiPe technique. This approach is noteworthy for its progressive nature, since it is the first to allow for the computation of both endpoint and midpoint indicators inside a single framework. Making use of this unique feature, the research blends endpoint and midpoint indicators according to what is thought appropriate for each situation. However, a more thorough investigation has been carried out, with a particular focus on improving the characterization of midpoint indicators, in order to reduce subjectivity and uncertainty^11–14^.

India uses a large quantity of energy each year, making it a country with a big energy consumption. But a significant amount of this energy, around 70.685 exajoules (EJ), is regarded as rejected energy, pointing to inefficiencies in energy use. It is imperative to switch to renewable energy sources like wind and solar power in order to solve this problem and satisfy rising energy demands in a sustainable manner. India would need to significantly boost its output of renewable energy, with the goal of producing at least 42.2 EJ a year, in order to replace conventional fossil fuels like coal, natural gas and petroleum. With the current energy environment mostly dependent on conventional sources, this poses a considerable hurdle^15–19^. The results of our model are in line with comparable conclusions in previous studies, despite the fact that capital costs show a great deal of fluctuation^20–23^. The consistency of our cost modeling technique with published results highlights its dependability and resilience when applied to electrical transmission infrastructure.

### Variations in pipeline capital costs due to assumptions about building site

The comparative life cycle assessment (LCA) and cost analysis of 33 kV electrical transmission lines and underground cables in India reveals significant variations influenced by construction location. Research literature indicates that urban areas incur higher costs due to expensive land acquisition, regulatory compliance and higher labor rates, while rural areas face increased costs due to challenging terrain, transportation logistics and specialized engineering requirements. Factors such as soil quality, weather conditions and security concerns further impact the costs. LCA studies highlight that overhead lines may have lower initial capital costs but higher maintenance requirements, whereas underground cables, though costlier initially due to excavation and material expenses, offer lower maintenance and reduced environmental and visual impacts. This assessment provides a holistic view of the environmental and economic trade-offs involved in selecting the appropriate transmission method for different regions in India. Table 3 in the cited literature presents a comparison of the fluctuations in capital expenses for several types of 36-inch diameter natural gas and oil. It emphasizes the influence of the building site on the costs. In the case of 33 kV electrical transmission lines and underground cables in India, the capital costs are greatly influenced by the construction location. Urban construction entails greater expenditures as a result of land procurement, regulatory obstacles, and labor expenses, whilst rural places encounter elevated costs due to challenging topography and logistical challenges. These factors result in significant fluctuations in the total capital expenses. Densely inhabited metropolitan zones have the highest potential prices, whereas less populous rural areas demonstrate the lowest conceivable costs. The significance of taking into account location-specific aspects in the development and execution of electrical transmission infrastructure in India is shown by this thorough cost analysis.

**Table 3:** Cost comparisons between capital cost models assuming pipeline construction in different geographies.

Energy source	Diameter	Model	Optimal budget zone (Capital cost)	Costliest area
Natural gas	38″	Bhardwaj et al.^13^	₹6596	₹9936
Natural gas	38″	Farnsworth et al.^14^	₹16,449	₹41,666
Oil	38″	Mensah et al.^15^	₹1419	₹2171
Oil	38″	Hafsa et al.^16^	₹3256	₹8099

This study utilizes a clearly delineated life-cycle framework to enhance methodological transparency for both environmental (LCA) and economic (LCCA) assessments. For the purpose of comparison, the functional unit is set at 1 km of 33 kV transmission line throughout a 30-year operational life. The system boundaries use a cradle-to-grave approach, which includes getting materials, making them, moving them, putting them together, using them and getting rid of them at the end of their existence. Authors used technical specifications from utilities and conventional engineering guidelines to build material inventories for conductors, insulators, poles, excavation work and XLPE cables. Authors got the emission factors for steel, aluminum, concrete and polymeric materials from reliable LCA resources. An environmental impact assessment was done using the ISO 14040/14044 standards. The examination of the life cycle cost looked at the costs of buying, installing, losing energy, doing normal maintenance, fixing problems and getting rid of the item at the end of its life. This precise analytical framework makes it easier to repeat tests and sets a standard for comparing overhead and subterranean 33 kV systems.

Inventory data were obtained from a combination of primary and secondary sources. Primary data such as conductor dimensions, pole specifications, excavation depths and installation energy were collected from utility-provided project documents. Secondary data such as emission factors for steel, aluminum, polymer insulation and concrete were sourced from the ecoinvent database and validated against published LCA studies. Table 4 summarizes the source type for each inventory element. Underground cable impacts (embodied materials, trenching emissions, operational losses and end-of-life treatment) are quantified numerically and presented alongside overhead system results to enable direct comparison. This transparent and fully traceable methodology ensures that all assumptions, boundaries and data sources are clearly defined and reproducible.

**Table 4:** LCA Results: 1 km of 33 kV line, 30-year lifetime.

Impact category (unit)	Overhead (OH) - total	Underground (UG) - total	OH - installation	UG - installation	OH - operation (losses)	UG - operation (losses)	OH - end-of-life (credit)	UG - end-of-life (credit)	Data source/note
Global warming potential (kg CO_2_-eq)	12,000	45,000	4000	30,000	7000	10,000	− 1000	− 5000	Example: OpenLCA + ecoinvent-like factors
Cumulative energy demand (MJ)	150,000	550,000	50,000	420,000	95,000	140,000	− 5000	− 10,000	MJ primary energy
Human toxicity (kg 1,4-DCB-eq)	200	800	60	600	120	200	− 10	− 100	Example midpoint indicator
Mineral resource depletion (kg Sb-eq)	0.45	1.6	0.12	1.3	0.25	0.45	− 0.02	− 0.60	Illustrative
Primary material mass (tonnes)	8.5 t	40.0 t	3.0 t	30.0 t	5.0 t (losses-related)	8.0 t	− 0.5 t	− 6.0 t	Shows larger material demand for UG
Embodied emissions per km (installation only) (kg CO_2_-eq)	4000	30,000	–	–	–	–	–	–	Highlights UG installation intensity

**Fig. 1:**
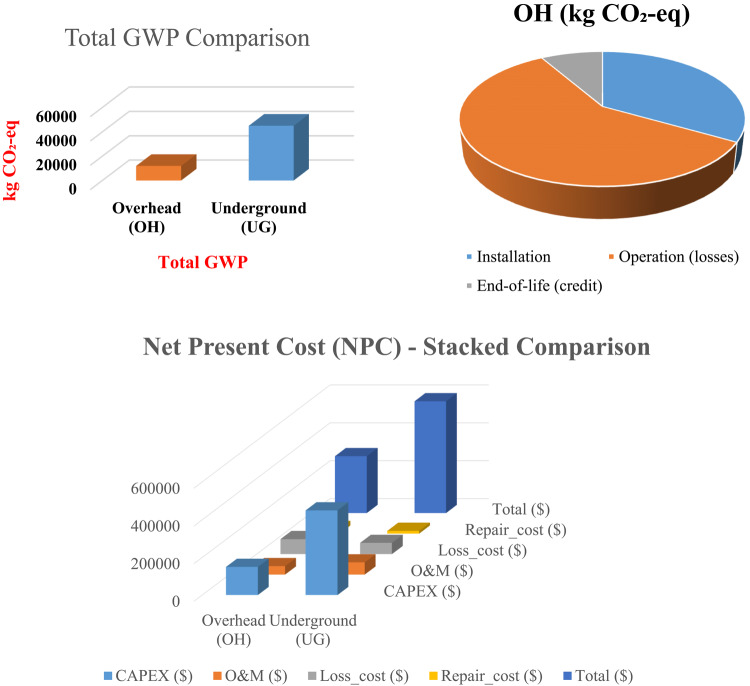
Comparative analysis: overhead versus underground systems.

### Functional unit and normalization framework

The primary functional unit of this study is defined as: Delivery of 1 kWh of electricity at 33 kV to the distribution node over a 40-year operational lifetime. All environmental impacts and economic costs are normalized to this functional unit.

#### Lifetime energy transmission

For a line of length $\:L$ (km) transmitting average power $\:P$*(MW)*: $\:{E}_{transmitted}=P\:\times\:8760\:\times\:40$  

#### Operational losses

Resistive losses are calculated as in Eq. 2:

2 $$\:{P}_{loss}={I}^{2}R$$

Total lifetime loss as in Eq. 3:

3 $$\:{E}_{loss}={P}_{loss}\:\times\:8760\:\times\:40$$

#### Net delivered electricity

4 $$\:{E}_{delievered}=\:{E}_{transmitted}-{E}_{loss}$$

All impacts are divided as in Eq. 5:

5 $$\:{Impact}_{perkWh}=\frac{Total\,\:lifetime\,\:impact}{{E}_{delievered}}$$

This ensures consistent normalization across environmental and economic metrics.

## Results and discussion

**Fig. 2:**
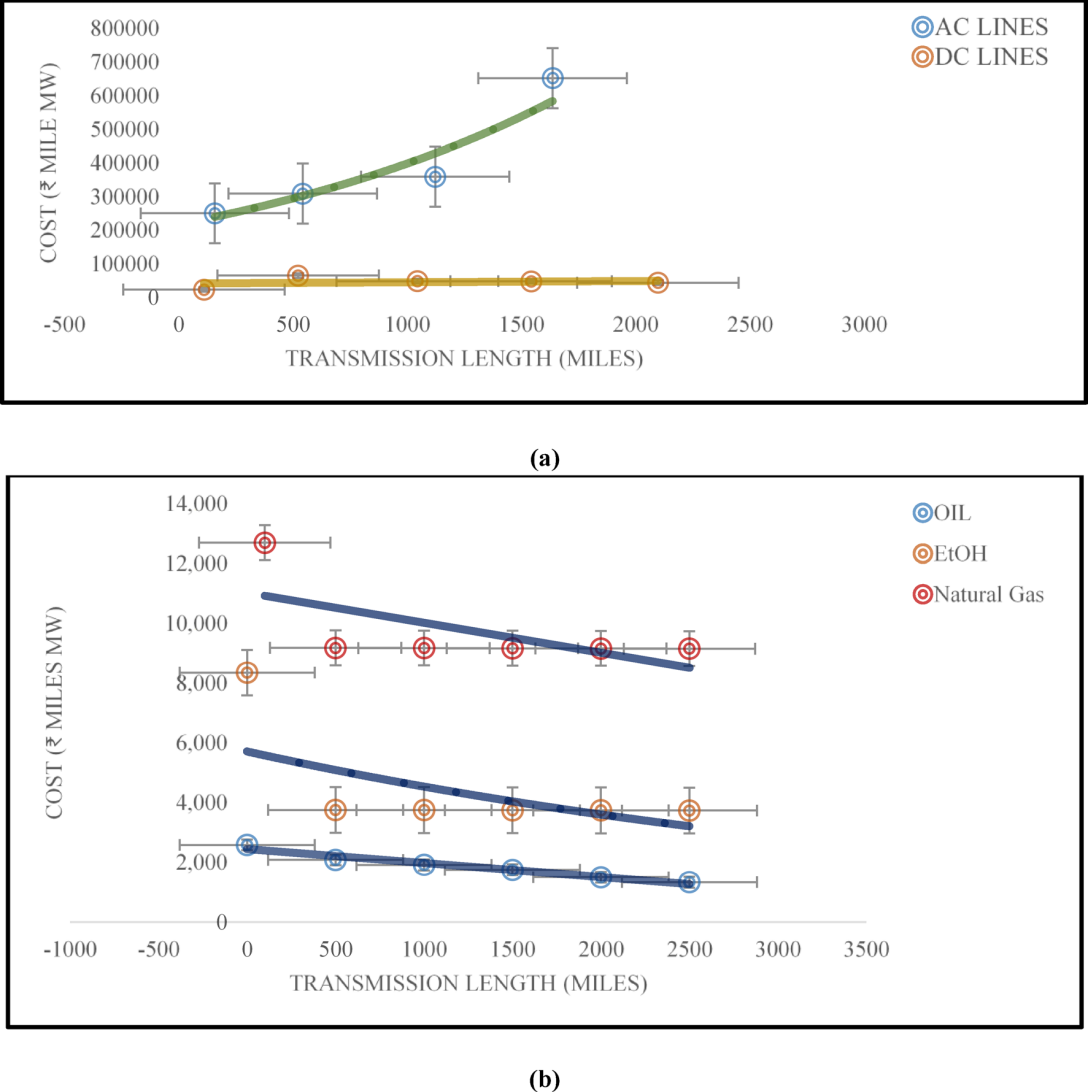
(**a**, **b**) Examining the relationship between transmission distance and price.

The cost dynamics of 33 kV electrical transmission lines and subterranean cables in India show patterns resembling those of pipeline transmission costs. Cost per unit of transmission tends to drop with increasing transmission line length. But, save for specific situations, the cost savings stop or become negligible beyond a certain distance, about 500 km. The cost categories connected to the infrastructure of electricity transmission reflect this tendency. For instance, over shorter distances material, labor and installation costs significantly drop but then level off over longer spans. Like pumping/compressor station expenses in pipelines, transformer and substation installations account for the largest portion of the overall cost and after early distances, exhibit significant declines. Remarkably, like pipeline transmission projects, the Right-of-Way (ROW) expenses stay the same no matter how long the project is. These cost trends highlight the need of strategic planning for the efficient optimization of costs in the extension of transmission infrastructure while considering the particular geographical and logistical difficulties that India faces as shown in Figs. 1 and 2a,b.

**Fig. 3:**
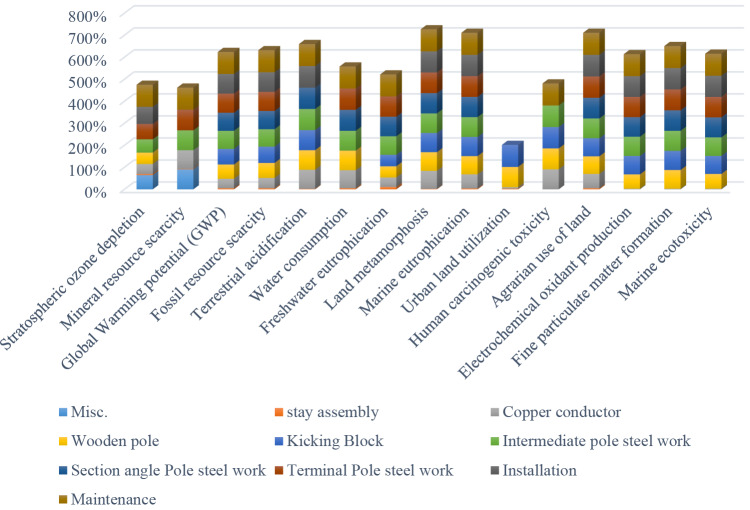
Elaborated effects of a 38 mm^2^ overhead line, spanning 1 km from the cradle to the place of disposal (LCIAM: ReCiPe midpoint).

**Fig. 4:**
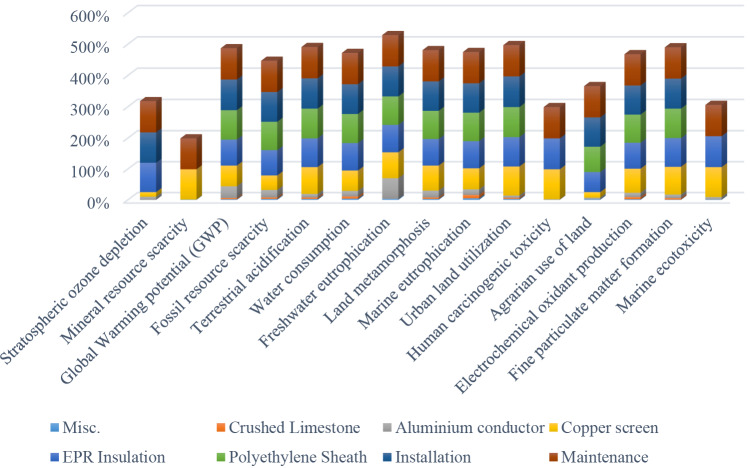
Elaborated effects of a 95 mm^2^ subterranean wire spanning 1 km from cradle to pre-disposal embedded (LCIAM: ReCiPe midpoint).

The ReCiPe midpoint method was used to conduct a comparative life cycle assessment (LCA) of 33 kV electrical transmission lines and underground cables. This assessment provides a thorough analysis of the environmental impacts at different stages, such as raw material extraction, manufacturing, transportation, installation, operation and end-of-life disposal. The Global Warming Potential (GWP) is higher for 1 km of underground cables compared to overhead cables due to the heavy use of materials and complex installation methods. In contrast, overhead cables have a lower GWP since they require less material and simpler installation as shown in Table 5. The depletion of fossil fuels has a notable impact on underground cables since their manufacturing and installation need a large amount of energy. In contrast, overhead cables consume fewer fossil fuels. Underground cables have a higher Human Toxicity Potential (HTP) due to the presence of abundant insulating materials and chemicals, in contrast to lower HTP in above cables. Underground cables have a higher Ozone Depletion Potential (ODP) because of certain insulation materials, while above cables have a lower impact on ozone depletion. Underground cables have a higher Acidification Potential (AP) compared to above cables because of emissions resulting from extensive building activity. The choice of materials for subterranean cables results in increased environmental implications in areas such as global warming potential (GWP), depletion of fossil fuels, and human toxicity potential (HTP). While underground cables do have a longer lifespan and require less maintenance, their initial environmental impacts are considerably greater. Overhead wires, which necessitate more frequent maintenance, have fewer initial environmental impacts. Recycling underground cables at the end-of-life stage can help reduce certain effects, however the process is typically more intricate and expensive. Although underground cables have advantages in terms of aesthetics and lower operational disruptions, their greater early environmental consequences must be carefully considered in relation to these benefits. This means that overhead cables may be more sustainable in terms of overall environmental performance during their lifespan. The graphical representation of the results for an overhead line with an area of 38 mm^2^ and an underground cable with an area of 95 mm^2^ can be seen in Figs. 3 and 4 respectively.

The operational ratings of these two cables − 130.5 A and 141.5 A (maximum normal loading) - were selected since they are somewhat similar. Copper conductors were often found to be the biggest contributor to embodied effects. Wooden poles show the next most significant contribution for the energy and carbon categories (biogenic carbon storage was not included). With 3.4 tonnes out of a total mass of 4.9 tonnes for the 25 mm^2^ cable and 4.3 tonnes out of a total mass of 9.1 tonnes for the 100 mm^2^ cable, wooden poles made up most of each overhead line assembly. In several impact categories, the “intermediate pole steelwork” also had a discernible effect. It specifically made at least 10% of the contributions to climate change, ozone depletion, freshwater eutrophication, freshwater ecotoxicity, ionising radiation, agricultural land occupation, water depletion and fossil depletion. It turned out that installing the overhead line was mostly unimportant. That cannot be said, though, of maintenance, which made a substantial contribution to a number of impact categories. Specifically, maintenance added 18% to the ozone depletion, 11% to the fossil depletion, 10% to climate change and 8% to the ionizing radiation. Weather-exposed and with a higher fault rate are overhead lines. The outcome is a maintenance schedule that calls for routine tree cutting and helicopter and Land Rover inspections.

**Table 5:** Impacts of 1 km of subterranean and overhead cables (LCIAM: ReCiPe).

Category of impact	Unit	25 mm^2^ O/H	38 mm^2^ O/H	100 mm^2^ O/H
Stratospheric ozone depletion	kg CFC-11 eq	0.00067	0.00093	0.00168
Mineral resource scarcity	kg Fe eq	35,612	53,722	135,022
Global warming potential (GWP)	kg CO_2_ eq	9767	12,567	23,934
Fossil resource scarcity	kg oil eq	2987	3765	7497
Terrestrial acidification	kg SO_2_ eq	278	391	992
Water consumption	m^3^	127	189	429
Freshwater eutrophication	kg P eq	0.697	1.07	1.96
Land use	m^2^	5.87	8.76	21.8
Marine eutrophication	kg N eq	10.7	14.6	32.6
Land use	m^2^a	27,160	27,720	41,557
Human carcinogenic toxicity	kg 1,4-DB eq	54,925	82,843	217,056
Agrarian use of land	m2a	227	324	727
Fine particulate matter formation	kg PM10 eq	91.5	133	330
Marine ecotoxicity	kg 1,4-DB eq	694	1050	2710

### Operational losses in 33 kV electrical transmission lines and underground cables

Operational losses in 33 kV electrical transmission lines and underground cables are primarily due to resistive, dielectric, inductive and capacitive losses. For overhead lines, resistive losses (I²R losses) occur due to the resistance of the conductors and can be calculated using $\:{P}_{loss}={I}^{2}\cdot R$, where *I* is the current and *R* is the resistance of the conductor. Dielectric losses in overhead lines are negligible, while inductive losses are influenced by the inductance *L* of the line, calculated as $\:{V}_{L}=L\cdot \frac{dI}{dt}$. Capacitive losses are lower in overhead lines due to the air insulation. In underground cables, resistive losses are higher due to different conductor materials and can be calculated similarly using $\:{P}_{loss}={I}^{2}\cdot R$. Dielectric losses in underground cables are significant and depend on the dielectric constant (ε) of the insulation material, calculated as $\:{P}_{dielectric}={V}^{2}\cdot \omega\:\cdot C\cdot \mathrm{t}\mathrm{a}\mathrm{n}\left(\delta\:\right)$, where (V) is the voltage, ω is the angular frequency, *C* is the capacitance, and $\:\mathrm{t}\mathrm{a}\mathrm{n}\left(\delta\:\right)$ is the loss tangent. Capacitive losses are more prominent in underground cables due to higher capacitance, *C*, calculated as $\:C=\frac{2\pi\:\epsilon\:}{\mathrm{l}\mathrm{n}\left(\frac{D}{d}\right)}$, where *D* is the distance between conductors and *d* is the diameter of the conductor. These losses can be mitigated through the selection of low-resistance conductors, optimized design and advanced technologies such as real-time monitoring and reactive power compensation.

### Particulate matter formation

Particulate Matter (PM), particularly with a diameter of less than 10 micrometers (PM10), represents a complex mixture of organic and inorganic substances, critical in evaluating the life cycle of 33 kV electrical transmission lines and underground cables. Primary particles are emitted directly during activities such as the construction and installation of these systems, involving dust from excavation and emissions from machinery. Secondary particles form through atmospheric chemical reactions involving sulfur dioxide (SO_2_), nitrogen oxides (NOx), and organic gases, often emitted during the manufacturing processes of cables and components. These pollutants react with atmospheric oxygen (O_2_), water vapor (H_2_O), and reactive species like ozone (O_3_) and hydroxyl radicals (·OH), leading to the formation of fine particulate matter. This PM can significantly impact local air quality and human health, contributing to respiratory and cardiovascular diseases. Therefore, understanding and mitigating PM formation is essential for the sustainable development and operation of electrical transmission systems.

Our investigation comparing 33 kV electrical transmission lines and underground cables has revealed significant differences in the generation of particulate matter (PMF) consequences. When conducting a comparison between a 100 mm² above line and a 185 mm² underground wire, the analysis uncovers fascinating patterns. At lower currents, specifically 50 A, the overhead line with a cross-sectional area of 100 mm² has a reduced life-cycle PMF impact per kWh compared to the buried line. Nevertheless, with the increase in current levels to approximately 125 A, the life-cycle PMF impacts come together, suggesting that both systems have comparable environmental footprints. After surpassing this limit, the subterranean cable measuring 185 mm² shows a reduced life-cycle PMF impact, mainly due to the greater PMF impacts involved in its production and installation stages. Surprisingly, although overhead lines have lower initial embodied impacts, they suffer from larger electrical losses throughout operation, leading to a higher life-cycle PMF impact per kWh compared to underground cables. The results emphasize the intricate relationship between building techniques, operating effectiveness, and environmental consequences while assessing 33 kV electrical transmission networks. When assessing long-term factors that contribute to the creation of Particulate Matter (PMF), it is essential to take into account the inherent uncertainties and ever-changing nature of energy markets. The composition of electricity supply undergoes regular changes, occurring even on a daily basis. Over time, modifications in power generation technology, such as the substitution of gas with wind or coal, can have a substantial impact on the mix of energy output. Therefore, it is essential to conduct scenario-based studies that take into consideration political, societal, economic and market fluctuations that can affect the accuracy of long-term predictions. This matter is frequently disregarded, as numerous studies depend on emissions variables generated from existing technologies. In authors investigation, authors utilized a comparable method, however authors encountered no issues due to the fact that both the 33 kV overhead lines and underground cables draw electricity from the same power source in the national grid. Given that conductor losses have been recognized as the main cause of environmental effect, any future modifications to the national electricity supply would have an equivalent impact on both transmission networks, thereby ensuring a consistent and fair evaluation.

Due to the ever-changing nature of power markets, which can change supply mixes, long-term PMF statistics should be read with care. The generating mix changes over time due to major transitions like switching from gas to wind or coal power, which calls for scenario-based evaluations that take market, social, political and economic volatility into consideration. Due to the identical electrical mix utilized by 33 kV overhead lines and underground cables, the results were unaffected by the application of emissions factors based on current technology in this study. The major environmental worry was conductor losses, not embodied consequences; this suggests that both transmission systems will be similarly affected by future changes in the nation’s electrical supply. In this study, the analysis determined that other variables were either insignificant or identical for both types of conductors. By comparing cables under the same loading conditions, current variations were excluded from consideration. Thus, the only significant factor remaining was conductor resistance. To minimize the environmental impacts of 33 kV cables, it is essential to select conductors with the lowest resistance. This choice reduces electrical losses during operation, which in turn lowers the overall life-cycle environmental impacts associated with the transmission system. It was shown in this study that other variables may be removed from the analysis since they were either not significant or were the same for both conductors. In order to test the cables, we used the same loading conditions so that we could stop thinking about current changes. Conductor resistance was thus determined to be the crucial component. Choosing low-resistance conductors is crucial for minimizing electrical losses during operation and lowering the environmental impacts throughout the life-cycle of the product. Deciding on the best cable option is made easier with this simplified method, which is vital for decision-makers. On top of that, it gives helpful information for thinking about installing a bigger conductor before the current one’s lifespan ends in order to further decrease cable losses.

### Integrated cost–environmental trade-off analysis

Both lifespan environmental impacts and lifecycle costs are heavily influenced by common technical criteria, particularly conductor material, cross-sectional areas, electrical resistance and installation layout. Larger conductor sizes may increase capital costs due to increased material use, but they also minimize operating I²R losses, lessening the environmental effect of electricity generation over time. Smaller conductors, on the other hand, have lower initial material costs but higher resistive losses during their operating lifetime, potentially raising cumulative emissions per delivered kWh. The study found that subsurface systems have higher embodied environmental impacts and capital expenditures due to insulating layers, trenching needs, and increased material intensity. However, in particular high-density metropolitan areas with high fault rates and maintenance disruptions, underground networks may show enhanced long-term reliability and lower outage-related indirect costs.

When normalized per kWh provided over a 40-year lifetime, operational transmission losses emerge as the most significant contributor to lifecycle environmental performance. As a result, conductor sizing becomes an important design element, influencing both economic and environmental effects. Optimizing conductor cross-section under Indian loading circumstances can dramatically cut lifetime emissions while being cost-effective. This integrated view indicates that infrastructure design decisions at the 33 kV level should not be based exclusively on installation cost comparisons but must also consider lifecycle loss models and environmental externalities as in Table 6.

**Table 6:** Key parameters affecting cost–environment trade-offs in 33 kV systems.

Design parameter	Cost impact	Environmental impact	Direction of influence
Conductor size	↑ Cost	↓ Losses	Trade-off
Resistance	↓ Cost	↑ Emissions	Negative
Underground trenching	↑ Cost	↑ Embodied emissions	Direct
Operational load	↑ Losses	↑ Emissions	Coupled

**Fig. 5:**
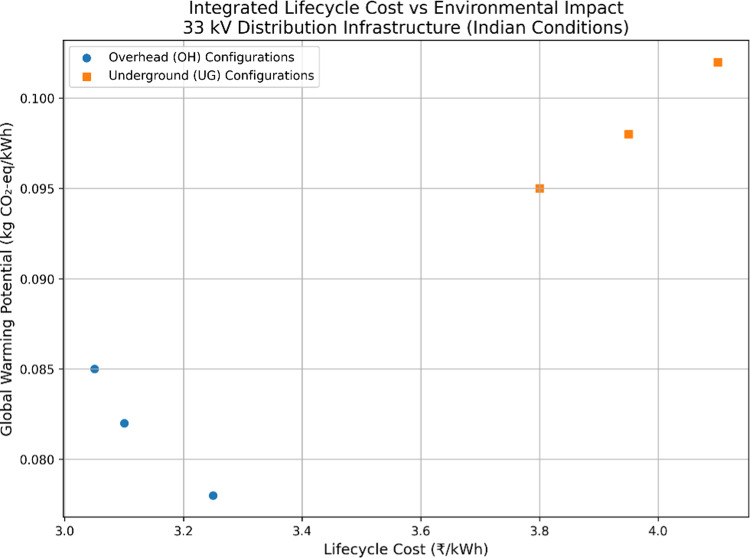
Integrated lifecycle cost–GWP comparison of 33 kV overhead and underground systems.

In Fig. 5, integrated lifecycle cost vs. global warming potential for 33 kV overhead (OH) and underground (UG) designs under Indian operating circumstances. The plot depicts the techno-environmental trade-offs that must be considered when making infrastructure decisions.

### Positioning of results relative to existing 11–33 kV studies

Existing research comparing overhead and subsurface distribution systems at medium voltage levels generally concludes that underground systems have 2–4 times greater installation costs and significant embodied environmental impacts due to insulating materials and trenching requirements. However, many of these studies are based on European material inventories, lower ambient temperatures and varying load profiles. In contrast, the current analysis includes Indian-specific characteristics such as conductor sizing procedures, greater ambient operating temperatures, and regionally representative electrical generating mix. Under these settings, operational I²R losses contribute more to lifespan GWP than embodied material impacts. This changes the relative importance of conductor resistance and load circumstances in infrastructure planning. Furthermore, the incorporation of lifespan cost normalization per supplied kWh provides a decision-relevant parameter that was not previously offered in 11–33 kV comparative studies[24].

The findings show that under Indian grid conditions, when the energy mix is still carbon-intensive, operational transmission losses dramatically increase lifecycle GWP. As a result, conductor sizing selections have a bigger environmental impact than systems operating with low-carbon power combinations. This distinguishes the current analysis from studies conducted in regions with lower grid emission factors.Multi-objective optimization methods used in microgrid scheduling also emphasize balancing cost and sustainability, which is important when comparing transmission options. [25]

### Limitations and future work

This study has a number of limitations that could affect how the results are understood. The LCA and cost estimates are based on guesses about things like how long parts will last, how often they need to be maintained, how far they need to be moved, and how good the data is in the databases that are available. Changes in the Indian electrical mix and differences in material production across regions further make things less clear. In the future, we will do more comprehensive sensitivity analysis to measure how parameters affect the results, add inventory datasets for specific regions, and expand the system boundaries to include recycling paths at the end of life. Adding probabilistic uncertainty assessment and checking conclusions against field data will make the method even stronger.

## Conclusion

This study provided an integrated Life Cycle Assessment (LCA) and Life Cycle Costing (LCC) evaluation of five 33 kV distribution options, comprising three overhead (OH) and two underground (UG) systems, under typical Indian operating circumstances. All impacts were constantly normalized per delivered kWh throughout a 40-year service life, allowing for direct techno-environmental comparisons between designs. The findings show that global warming potential (GWP), fossil resource depletion, and particulate matter generation are the most significant environmental effect categories. In all designs, operational I²R conductor losses are the largest contributor to lifecycle environmental burden per provided power. This research emphasizes conductor resistance and loading conditions as crucial design parameters that influence both environmental performance and lifecycle cost. The integrated cost-environmental study shows a clear techno-economic trade-off. Underground systems have higher embodied material impacts and capital expenditures due to insulation layers and installation intensity, whereas overhead systems have lower initial material burdens but are still susceptible to operational loss-driven impacts over long service lifetimes. When evaluated as a complete functional unit, conductor optimization appears as a major technique for decreasing lifecycle emissions while enhancing cost efficiency. Importantly, the dominance of operational loss impacts is amplified under Indian grid emission factors and loading practices, distinguishing the present findings from prior medium-voltage studies conducted under lower-carbon electricity mixes. This underscores the necessity of region-specific lifecycle normalization in infrastructure planning. Overall, the study provides a transparent and decision-relevant framework for evaluating 33 kV distribution infrastructure, supporting utilities and policymakers in balancing environmental performance, economic viability and long-term system efficiency. Future research may extend this framework through uncertainty analysis, dynamic load modeling and assessment of emerging low-loss conductor technologies.

## Data Availability

The datasets used and/or analysed during the current study are available from the corresponding author on reasonable request.
